# The Birth Companions’ Experience of the Birthing Room and How It Influences the Supportive Role: A Qualitative Study

**DOI:** 10.1177/19375867231163336

**Published:** 2023-04-27

**Authors:** Helena Nilvér, Marie Berg

**Affiliations:** 1 Institute of Health and Care Sciences, Sahlgrenska Academy, University of Gothenburg, Sweden; 2 Faculty of Medicine and Community Health, Evangelical University in Africa, Bukavu, Democratic Republic of Congo

**Keywords:** birthing room, birth companion, Sweden, labor and birth, physiological birth

## Abstract

**Aim:**

To explore birth companions’ experience of the birthing room and how it influences their role supporting the woman during labor and birth.

**Background:**

Although support from a birth companion positively affects the outcome of labor and birth, limited research explores how the birthing room influences the companion. This study identifies elements of the birthing room essential for the birth companion to offer optimal support to the woman during labor and birth.

**Methods:**

Fifteen birth companions were individually interviewed 2 weeks to 6 months after birth using a semi-structured interview guide. Transcribed interviews were analyzed based on reflexive thematic analysis.

**Results:**

The findings are captured by one overall theme: creating a supportive birth space in an unfamiliar environment. This creation process is further described by three subthemes: not being in the way, finding one's role, and being close to the birthing woman.

**Conclusions:**

The findings illustrate how the birthing room was an unfamiliar environment for the birth companions, but one that they needed in order to give the required support. With slight changes in physical design, the birthing room can become calmer and more private and better help the birth companion fulfill the supportive role.

## Introduction

This article examines birth companions and their roles supporting women during labor and birth. A birth companion should be chosen or accepted by the birthing woman and can be the father to be or a partner, relative, friend, doula, or healthcare professional. Support from a birth companion includes providing information, advocacy, practical and emotional support, and being continuously physically present (Bohren et al., 2019). A systematic literature review found that women receiving continuous support from a birth companion experienced healthier outcomes in terms of being more likely to have spontaneous vaginal births and fewer caesarean sections, less likely to report negative feelings about childbirth, less likely to use pharmacological analgesia, and less likely to have a baby with a low 5-min Apgar score. In addition, the duration of labor was likely to be shorter. The effect was greater if the birth companion was not a professional associated with the healthcare facility (Bohren et al., 2017).

Birth is a neurohormonal physiological and psychological process, particularly influenced and controlled by oxytocinergic mechanisms. This process is influenced by sensory stimuli, for example, from the uterus and by one-to-one support offered by a birth companion. Also, the external environment might influence these neurohormonal processes. A stressful and unfamiliar environment activates the sympathetic nervous system and inhibits the release of endogenous oxytocin, leading to weakened or ceased labor contractions, while a calm, private, and safe environment reduces stress and increases the release of endogenous oxytocin (Olza et al., 2020; Uvnas-Moberg et al., 2019). However, scientific evidence regarding the birth environment, especially the birthing room, and its effects is sparse (Nilsson et al., 2020; Setola et al., 2019).

In the Swedish Room4Birth research project, we found that the birthing room, through its design, could support either a salutogenic physiological birth process or a pathological treatment of childbirth (Andrén et al., 2021; Goldkuhl et al., 2022). We also found that birthing rooms adaptable to the needs of the birthing women helped them feel welcomed and supported, resulting in a more positive birth mood (Skogström et al., 2022), and that women cared for in more personally adapted rooms expressed a greater sense of safety, control, and integrity than did women being cared for in regular birthing rooms (Goldkuhl et al., 2022). If the birth companion had sufficient space and felt comfortable in the birthing room, this helped the birthing woman relax and focus on giving birth (Skogström et al., 2022).

There is limited research exploring birth companions’ experience of the birthing room design. A video ethnographic single-case study in Australia found childbirth supporters to be in a physical space that did not facilitate their presence and roles (Harte et al., 2016).

***There is limited research exploring birth companions’ experience of the birthing room design.***

This study explores the birth companions’ experience of the birthing room and how it influences their role supporting women during labor and birth. The knowledge gained is intended to identify design elements of the birthing room essential for birth companions to offer optimal support to women during labor and birth. The study is part of the Room4Birth research project, which aims to expand evidence-based knowledge of the design of the maternal healthcare environment and how it influences maternal and newborn health and care outcomes (https://www.gu.se/en/research/room4birth).

## Method

To capture birth companions’ experience, we chose a qualitative approach. Interviews and reflexive thematic analysis (Braun & Clarke, 2006, 2021a) were used to identify meaning patterns associated with the research question.

### Study Setting and Participants

**Figure 1.**
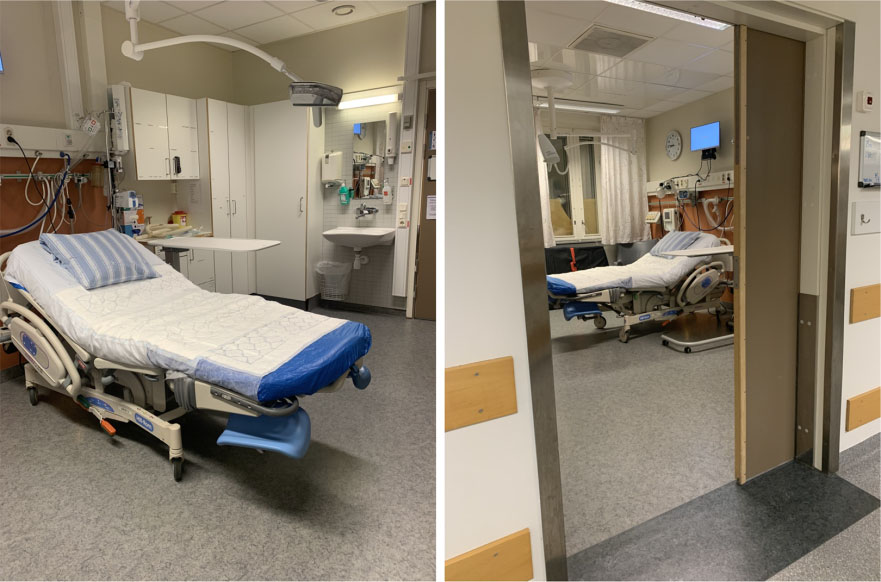
Typical birthing room at the recruiting hospital. *Source*: Photos by Lisa B. Skogström.

Almost all births in Sweden occur in hospitals, with only about one per 1,000 being planned home births supported by midwives (Svenska Barnmorskeförbundet [The Swedish Association of Midwives], 2020). To capture a wide range of experience, we included a variety of birth companions with different birth support experience. Study participants were recruited through (i) a university hospital, (ii) a transcultural doula organization supporting pregnant women who do not speak Swedish, and (iii) a midwife team assisting at home births. The hospital had three labor wards situated near neonatal, surgical, and intensive care units. Women in active labor were cared for in single birthing rooms, each with a toilet and equipped with medical technology such as a computer for keeping medical records, a monitor for fetal cardiotocographic monitoring, nitrous oxide equipment, and a drop counter. The birthing bed was placed in the center of the room. The birthing rooms all had an armchair and a birth ball, some rooms had a sofa, and a few rooms had a bathtub. All rooms were equipped with an alarm system to summon help, which also indicated if there was an alarm from any other rooms in the ward. Hospital policy allowed two birth companions to be present in the room. A typical birthing room is shown in Figure 1.

When all birthing rooms were occupied, the women could be cared for in small “arrival rooms” in which the toilet was not partitioned off. In home births, women arranged their birth environment according to their own wishes. Most of them rented a birthing pool, which was placed in the living room and where the birth often took place. In addition, most home births were attended by two midwives hired from a private midwifery organization.

The study participants were recruited by a contact midwife at the hospital, the leader of the transcultural doula organization, or a midwife coordinating the midwives’ home birth team. Eligible birth companions obtained oral and written study information from the contact midwife or one of the researchers (H.N.), and those consenting were contacted by H.N. 1 week after birth by email or phone. They were informed that participation was voluntary, that they could withdraw consent at any time, and that the data would be handled confidentially. All participants signed a consent form before an individual interview scheduled according to the participant’s availability.

A total of 15 birth companions were individually interviewed: eight fathers of the child being born, four doulas (of whom three were transcultural), two relatives, and one friend. Characteristics of the study participants are presented in Table 1.

***A total of 15 birth companions were individually interviewed: eight fathers of the child being born, four doulas (of whom three were transcultural), two relatives, and one friend.***

**Table 1.** Characteristics of the Study Participants.

No.	Age	Education	Country/Area of Birth	No. Attended Births	Relation to Birthing Woman	Birthplace
ID 1	30	University	Europe outside Nordic countries	1	Partner	Hospital
ID 2	32	High school	Sweden	2	Partner	Hospital
ID 3	30	University	Sweden	1	Partner	Hospital
ID 4	43	University	Sweden	>100	Doula	Hospital
ID 5	39	University	Middle East	3	Partner	Hospital
ID 6	32	High school	Africa	2	Partner	Hospital
ID 7	31	University	Sweden	1	Partner	Hospital
ID 8	33	University	Sweden	2	Partner	Hospital
ID 9	46	High school	Africa	>100	Doula	Hospital
ID 10	46	University	Sweden	3	Partner	Hospital
ID 11	58	High school	Africa	ca. 80	Doula	Hospital
ID 12	35	University	Europe outside Nordic countries	1	Relative	Hospital
ID 13	47	High school	Middle East	>100	Doula	Hospital
ID 14	34	University	Sweden	1	Friend	Home birth
ID 15	58	High school	Sweden	1	Relative	Home birth

### Interviews

The interviews were based on a semi-structured interview guide (Supplement Material 1) tested and developed in two pilot interviews with birth companions. The interviews were informal with the companions first being asked to describe how they perceived the birthing room with follow-up questions such as how the room affected them, if they felt comfortable and if there was a space for them. Thereafter, questions were asked to cover topics such as if they felt free to adapt the room to their and the birthing woman’s needs, parts in the environment that were important for them, and elements of the birthing room that facilitated or distracted them in their supportive role. All interviews were conducted by H.N., one in a secluded room at the researchers’ office and all others using a video communication tool (zoom.us). All were audio-recorded and lasted 36 min on average; an independent agency transcribed the recordings.

### Analysis

Reflexive thematic analysis is a flexible method comprising several steps (Braun & Clarke, 2006, 2021a). The analysis was conducted jointly by both researchers (H.N. and M.B.). First, we read the interviews to become familiar with their content. The data were then coded in subsequent repeated readings. These codes organized the data and enabled them to be sorted into potential themes and subthemes. Next, the themes were reviewed and refined through reflecting on the interview data to ensure that no potential themes had been missed. Finally, the results were written up and further refined until the narratives in the data were conveyed in a concise, logical, and nonrepetitive way.

In reflexive thematic analysis, the subjectivity and reflexivity of the researcher are important tools in the research design, enabling the analysis to be richer and more insightful (Braun & Clarke, 2021a). Both of us researchers (H.N. and M.B.) are midwives with long experience of working in labor wards and of conducting qualitative interview studies. Throughout the analytical process, we strove to ensure quality and rigor in the analysis through moving back and forth between the interview data and tentative themes. We also discussed our subjectivity, reflecting on the thoughts and insights it gave us and how it influenced our interpretation of the data. This reflective stance broadened and nuanced the analytical perspective.

### Ethical Considerations

The study was designed and conducted in line with the Declaration of Helsinki: Ethical Principles for Research Involving Human Subjects (World Medical Association, 2013). Ethical approval was granted by the Swedish Ethical Review Authority (dnr. 2022-03737-01).

## Results

The birth companions’ experience of the birthing room and its influence on their ability to support the women during labor and birth are captured by one overall theme: *creating a supportive birth space in an unfamiliar environment.* This creation process is further described by three subthemes: *not being in the way*, *finding one’s role*, and *being close to the birthing woman*.

### Creating a Supportive Birth Space in an Unfamiliar Environment

The overall theme, *creating a supportive birth space in an unfamiliar environment*, reflects the fact that, for the birth companions, the birthing room was an unfamiliar environment where they had to strive to give support during childbirth. The companions were dependent on the labor ward staff and without their own place in the room, feelings of loneliness and of being an outsider developed. The presence of medico–technical equipment in the room reinforced this feeling. A medical risk perspective dominated, subordinating the role of the birth companion, and to offer support, it was important not to be in the way, to find one’s role, and to have opportunity to be close to the birthing woman. To be optimally supportive of the birthing woman throughout the birth process, the birth companions had to feel comfortable, relaxed, and be free to move.

***A medical risk perspective dominated, subordinating the role of the birth companion, and to offer support, it was important not to be in the way, to find one’s role, and to have opportunity to be close to the birthing woman.***

## Not Being in the Way

The theme *not being in the way* describes the birth companions’ striving not to be in the way in the birthing room, which, in its physical design, indicated that giving birth is a risky event that requires specialized knowledge and technology. To be in such a birthing room was to be in someone else’s world, and this made the companions uncertain about how to behave and afraid of being in the way. Therefore, the companions found it natural to stand aside and leave room for the labor ward staff, who had the required knowledge to manage the risky birth event in this technocratic environment:

When you could follow what was happening and understood the rules of the room, like where my partner’s place was and where the midwife would be.…I didn’t want to be in the way of the midwife or the nurse. When I entered the room, I didn’t go to my partner’s side, because I didn’t want to be in the way, but let the professionals do their job. (ID 1)

For some birth companions, the medico–technical equipment in the birthing room engendered a feeling of safety. It ensured that the room was a safe place where any medical complication that might arise during the birth process could be handled. For other birth companions, the medical equipment signaled that birth was not a normal physiological process that the woman’s body could handle, prompting worrying questions concerning when and how the equipment might be used and what types of emergencies could happen:

There were a lot of tubes. It really looked like a hospital environment. Everybody says, “You are not sick—this is health care [to have a baby].” But it did not feel like that. Or at least the room didn’t say so. The room instead said, “Here we can resuscitate you, in case you die,” sort of. (ID 3)

The size and furnishings of the birthing room indicated planning for the presence of only one birth companion. So as not to be in the way, the companions needed their own space where they could put their belongings without hindering staff in their jobs. The windowsill, sofa, and/or chair were such spaces, whereas the areas around the computer, cabinets, and wall with medical equipment were spaces the birth companion avoided so as not to hinder the labor ward staff in their work. When settled in a dedicated chair, the companion could feel more relaxed:

It felt like it [i.e., the armchair] was my place, this is where I belong. You are so lost as a partner. What should I do? How am I not in the way of all these important people who will do all these important things so that my baby doesn’t get hurt, etc. (ID 8)

The birthing bed was usually placed in the center of the birthing room, indicating that this was where the main event would happen: It was to be used by the woman during labor and was where the baby would be born. The bed was technical and difficult for the birth companion to adjust, and it was covered with plastic, indicating that the birth could become messy:

It [i.e., the bed] was like the centerpiece of the room. It’s what catches your eye when you enter the room. You look at the bed and you see the list behind it with all the pipes, tubes, and regulators. You understand someone is going to lie there, and it probably won’t be nice. (ID 3)

It was important for the birth companion not to disturb the labor ward staff unnecessarily, as they seemed very busy and stressed. It was considered logical that the staff would not be continuously present but generally only come into the room as needed and then leave. When help was needed, the birth companions had to call using the buzzer, which was a secure link to the staff:

I felt we could manage here. And then there was the buzzer, so I didn’t feel alone, which was nice. (ID 3)

## Finding One’s Role

The subtheme *finding one’s role* describes the birth companions’ attempt to find their role in an unfamiliar environment and culture, with the risk atmosphere signaled by the birthing room and the whole labor ward. This made them feel lost and uncertain about how to behave. As expressed by one birth companion, being in the birthing room gave a feeling of being abroad in a foreign country:

It is sort of like visiting another country—you don’t really know the culture and how to behave, because you haven’t been to this country before…. I didn’t know, what were my rights as a partner? Do I get something to eat, or do I need to go and buy it myself? And in that case, how do I get back in, as it [i.e., the door to the labor ward] was locked. I, who had never been there before, had no clue. Where do I go to the toilet? Can I sleep somewhere? Can I even stay? (ID 8)

While caught up in the labor and birth process in this unfamiliar environment, the birth companions tended to neglect their own needs, such as resting, eating, and using the toilet. They were often caught up in the birth process, and prioritizing their own needs was difficult as the birthing woman was central. On the other hand, they needed to fulfill their basic needs to have energy for the supportive role, but it was difficult to do this in the birthing room. One birth companion described how, during a longer labor, she rested in an armchair, covering her head with a scarf to ensure privacy, but still could not get needed rest. Eating was also difficult or almost forgotten, or they just grabbed convenient food such as a banana. Another birth companion expressed:

It worked for me as I knew it was a temporary thing. I wouldn’t be there for more than 1 or 2 days. I knew my wife would have a much more difficult time, so I shouldn’t complain about a mattress [on the floor]. I was happy just to have a place to sleep. (ID 7)

Another example of how the birth companion’s needs were marginalized was the lack of opportunity to use the toilet in privacy. The toilet in the birthing room could be occupied by the birthing woman and be messy, and, if not shielded by a door, it did not invite the birth companions to use it:

It was a toilet without a door, just partitioned off with a room divider. So, it wasn’t a private situation to use the toilet. After a brief “knock, knock,” someone could come into the room and just stand there. Hm, so it felt a bit uncomfortable for us both if you needed to use the toilet. (ID 10)

When caught up in the birth process, one could lose awareness of time and space. The birth companions could perceive themselves as confined in the birthing room. Looking through a window could give them a sense of time and space, as it reminded them of the world outside the birthing room. Rain spattering on the window, a sunset or sunrise all helped them relate to the outside world. More space and a window with a view could mitigate the experience of the birthing room as a bunker:

Well, it was sort of like a bunker. There were a lot of things in the room, hence the strained feeling. And the window, there was a building directly outside of it. It felt very crowded. (ID 12)

## Being Close to the Birthing Woman

The theme *being close to the birthing woman* captures the closeness to the birthing woman the birth companions needed in their supportive role. Being close was important so that the woman could feel that the companion was there for her, and this entailed both mental and physical closeness. Physical closeness could, for example, entail holding a hand, giving a massage, and having eye contact, while mental closeness implied creating an atmosphere of intimacy in the room:

I believe that distance can feel very great when giving birth. Even if it is just half a meter, it can sort of feel like “Where are they?” So I try to be as close as possible. With good eye contact to show “you are doing well.” And by using my hands a lot, through touch, I need to be close. (ID 4)

However, the physical design of the ordinary hospital birthing rooms, with their medico–technical equipment, did not invite such closeness, but rather tended to distance the birth companion from the birthing woman. For example, the electronic monitor of contractions and fetal heartbeats could draw attention away from the birthing woman’s experience of contractions. Observing the baby’s heart rate on the screen of the electronic fetal monitor could contribute to a sense of safety as long as everything was normal, but if the curve became unusual and attracted attention from labor ward staff, it instead became a stressor:

It gave some control [the electronic fetal monitor]. You imagine you have control, but you actually don’t. But it gave some form of security. It is on 130 now, super, or 120. At some point it went down to 80 and then my partner changed position and it went up again. That felt so good. (ID 1)

The centrally located birth bed was large, but not large enough to be used by two people, such as the woman and her partner. The complex technical design of the bed made the birth companion dependent on the staff to adjust it, so having their eyes at the same height as the birthing woman’s eyes was difficult. They described how they struggled to gain closeness at the right height by leaning over the bed in strange positions, kneeling, and positioning the armchair near the bed. In contrast, the home birth setting enabled closeness and intimacy in a more natural way:

I don’t know if it was the room that enabled us to be so very close to each other. Just that we had the couch where everyone could fit, the whole family, and the [birthing] pool where she [i.e., the birthing woman] could sit towards the side, and everyone could touch each other, and it was natural that we all wanted to be close. (ID 14)

Being close required that the birthing room had an atmosphere of privacy, intimacy, and calm. The birth companions found the labor ward staff to be central to creating such an atmosphere by, for example, respecting the privacy of the birthing woman, protecting her from exposure, not going into and out of the room unnecessarily, and knocking before entering. In a more homely and private environment, it was easier for the birth companions to be and feel close to the birthing woman. A homely environment enabled a more intimate atmosphere, which could be created by adding color, providing dimmable lighting and soft music, and hiding medical equipment. Birth companions with experience of assisting at both hospital births and home births concluded that such an atmosphere was easier to create in the home birth setting:

I was kind of scared, or worried about it before. But it turned out so well, and it was really because it was such a calm environment, with calm and harmonious people. The two midwives were absolutely amazing. They were low key, staying in the background and acting only when there was a need for support or to do something. One of them was knitting between contractions, and that contributed to the sense of calm. (ID 15)

There were no other voices, just the family and me talking. The midwife stayed in the background, so there were no other interrupting features. It felt a bit like we were in a bubble—it was very special. (ID 14)

***the physical design of the ordinary hospital birthing rooms, with their medico–technical equipment, did not invite such closeness, but rather tended to distance the birth companion from the birthing woman.***

## Discussion

This study, exploring birth companions’ experience of the birthing room and how it affects their role supporting the birthing woman, shows that it was a challenge to create a supportive birth space in the birthing room, which appeared as an unfamiliar environment. This challenge simultaneously entailed not being in the way of labor ward staff and finding a role in the room that enabled closeness to the birthing woman, which was essential to her support.

***This study, exploring birth companions’ experience of the birthing room and how it affects their role supporting the birthing woman, shows that it was a challenge to create a supportive birth space in the birthing room, which appeared as an unfamiliar environment.***

### Strengths and Limitations of the Study

In reflexive thematic analysis as conducted here, reflection on the “information richness” of the data set is recommended (Braun & Clarke, 2021b; Malterud et al., 2016). Our data set, containing individual interviews with 15 birth companions, was large and captured a rich diversity of experience sufficient to answer the research questions. We used an interview guide, enabling us to cover various topics and elicit detailed descriptions of the birthing room from the participant’s experience. That we interviewed birth companions with different functions and different relationships with the mother-to-be is another strength. Partners, relatives, a friend, and doulas (three with experience of being birth companions at home births) gave us a rich variety of experience to draw on and thus a robust analytical basis. A limitation was that all participating birth companions were recruited from one hospital with three labor wards, limiting the range of considered experience. It was initially our plan and would have been advantageous also to include participants from smaller hospitals; however, no relevant contact person came forward to identify tentative interviewees from these settings. This study was conducted in Sweden, so further research on birth companions’ experience of the birthing room in other national contexts is needed to strengthen the results.

### Creating a Supportive Birth Space in an Unfamiliar Environment

Our finding that birthing rooms of medico–technical design were experienced as unfamiliar is unfortunately aligned with what hospital-based labor wards generally provide (Stenglin & Foureur, 2013). The birth companions described needing to adapt to this unfamiliar environment—sometimes described as a foreign world—as a precondition for being present and giving support during the generally long-lasting labor and birth. This is aligned with qualitative findings from the Australian study showing that, according to birth companions, the design of the birthing room evoked an “unbelonging paradox,” implying that the companions felt unwelcomed and under-supported and that the room hindered their support (Harte et al., 2016).

The theme *not being in the way* captures how the birth companions tried to avoid interfering with the labor ward staff. The technocratic model of medicine has been described as a healthcare paradigm that requires, for example, the following of certain routines during the labor and birth process, which is assessed as inherently risky and in need of control by medical and technological expertise (Davis-Floyd, 2001). That birth is viewed as “medical” and risky was confirmed in a study using birthing room images, in which a single electrically powered hospital bed was centrally positioned with medical equipment around it and mediating its importance (Bowden et al., 2016). This medico–technical approach to the birthing room was what the birth companions in our study expected and experienced. They described the room as “someone else’s world,” where they needed to be out of the way of the labor ward staff who have the required knowledge to handle the risky labor and birth process. This confirms what has been concluded in earlier studies, that is, that the normal physiological process of childbirth is usually not supported in modern hospital-based labor wards (Bohren et al., 2017). Nevertheless, providing such support is crucial, as childbirth is a sensitive physiological process also influenced by environmental factors (Olza et al., 2020; Uvnas-Moberg et al., 2019). The importance of the birth companions having their own space has been shown in earlier studies within the Room4Birth project (Andrén et al., 2021; Nilsson et al., 2020; Skogström et al., 2022). In contrast, the interviewed birth companions who were able to function in a homely birthing room environment did not have to be concerned about being in the way of the assisting midwives, so they could relax and focus on their supportive role.

The theme *finding one’s role* captures how the birth companions felt lost and tended to neglect their basic needs. Previous research has shown that fathers want to be active participants in their partners’ labor, and that being a calm and supportive birth companion can be emotionally challenging (Johansson et al., 2015). However, our study presents examples of how the birthing room design quite easily could be adapted to provide a more relaxing environment for the birth companions, helping them find their role, see to their own needs, and thereby be able to give optimal support.

The theme *being close to the birthing woman* emphasizes the closeness the birth companions needed as part of their supportive role. However, the birthing room design instead tended to distance them from the birthing woman, not promoting the privacy and integrity that they needed to secure for the birthing woman. A scoping review has described how the birthing room could be perceived as more private and intimate if a small area separates it from the corridor, indicating a gradual transition from outside to inside, and the bed is positioned to create a sense of intimacy (Setola et al., 2019). In our study, the medical equipment in the room was another aspect found to distance the birthing companion from the birthing woman. Another study in the Room4Birth project showed that hiding medical equipment behind wooden panels could lessen stress and contribute to a more familiar birthing room environment (Skogström et al., 2022). The results in our study showed the need for a birthing room design that facilitates physical closeness between the birth companion and the birthing woman, as the companion often struggled in unergonomic positions.

The birthing room, apart from the physical space, is found to be the site of human interactions that affect the atmosphere in the room (Goldkuhl et al., 2022). This was confirmed by our findings, especially regarding the theme *being close to the birthing woman*, which concerns both physical and mental closeness. Mental closeness implied creating an atmosphere of calm intimacy in the room. The labor ward staff were part of the birth environment and could contribute to this atmosphere. In a midwifery model of woman-centered childbirth care, developed in studies in Sweden and Iceland, one of the three central and intervening themes in the model is to create “a birthing atmosphere” that fosters feelings of calm, trust, and safety and that supports a sense of normalcy. The midwife has a central role in creating such a birthing atmosphere, but in a hospital-based birth environment, this is a great challenge (Berg et al., 2012) due to the dominance of a medico–technical perspective both in guidelines and in the presence and extensive use of medico–technical equipment (Andrén et al., 2021; Davis & Homer, 2016). For midwives, it is therefore a major challenge to create a positive space for childbirth (Andrén et al., 2021; Berg et al., 2012; Davis & Homer, 2016). The midwife’s presence can nevertheless help make the room seem more private by, for example, protecting the birthing woman from disturbing features inside and outside the room. By their calm presence and body language, midwives can convey that everything is normal (Andrén et al., 2021). This was confirmed in our study, in which we showed how the midwives by their continuous and calm presence in the background—only stepping forward when there was a need for extra support or attention—was by the birth companions experienced as contributing to a sense of safety and calm.

## Conclusion With Implications for the Birthing Room Environment

The birth companion’s support is important for the birthing woman and positively influences her childbirth experience as well as the outcome of labor and birth (Bohren et al., 2017). By exploring the birth companion’s experience of the birthing room and how it affects their role supporting the birthing woman, we showed that it was a challenge for them to create a supportive birth space in the birthing room as it was an unfamiliar environment, but with slight changes the birthing environment can be more homely and private, and allowing the birth companion to find “space” to fit in. The ideal is that the room should be designed so that it can be changed and adapted to personal wishes and needs of both the birthing woman and her birth companion(s). Such adaptations can be jointly made by the birthing woman, the birth companion, and the midwife or other staff.

Based on our findings, we have made a list of recommendations for aspects of birthing room design that facilitate birth companions in their supportive role during labor and birth. These recommendations can be considered both in adapting existing birthing rooms and constructing new ones.

- Provide storage space for the birth companion’s and birthing woman’s belongings—This would create a welcoming feeling by showing that both have important roles in the birth.
- Create sufficient space for the presence of several birth companions—The birthing room needs to be spacious enough for several birth companions.
- Provide comfortable furnishings so everyone can sit and rest—These should include both a large sofa and movable armchairs to enable closeness to the birthing woman.
- Hide the medical equipment—Although this equipment can provide a sense of security, it also signals that birth is risky and can raise worrying questions that disrupt the birth companion in their supportive role.
- Provide a homely environment that is soft and cozy and allows the birth companion to personalize the room—The features should include soft and dimmable light and the ability for individual choice of music.
- A spacious bed should be positioned carefully to provide privacy—A hospital bed positioned centrally in the room signals to the birth companion that this is where the baby is to be born, discouraging movement during labor and birth. Also requested was a bed large enough for both the birthing woman and her companion, so the companion can provide support through physical closeness.
- Provide clear descriptions of where the birth companion can conveniently obtain beverages and snacks nearby—This would enable the companion to get refreshments while remaining near the birthing woman.
- Ensure that there is a window with a view that allows the amount of daylight to be controlled—There is a need for a window that the birth companion can look out to follow the rhythm of the day and changes in weather, so as not to lose track of time. The window should be positioned to ensure privacy but allow the amount of daylight entering the room to be controlled.
- A small hallway between the birthing room and the corridor would constitute a transitional space for those entering the birthing room, thereby increasing privacy.
- An additional toilet is needed in the labor ward for the birth companions—The toilet in the birthing room might well be occupied by the birthing woman, and an additional toilet outside of the birthing room would give the birth companion some privacy.
- A quiet and peaceful space outside of the birthing room where birth companions could briefly rest—This should be separated by a door from the waiting area or day room, as these areas can become crowded and do not provide the privacy needed for rest.

## Implications for Practice

- Our study found the birthing room design tending to distance the companions from the birthing woman, not promoting the privacy and integrity that they needed as part of their supportive role. There is a need for a birthing room design that facilitates physical closeness between the birth companion and the birthing woman.
- The ideal is that the room should be designed so that it can be changed and adapted to personal wishes and needs. Examples are presented of how the birthing room design could be adapted to provide a more relaxing environment for the birth companions in which they can find their role and place, fulfill their needs, and thereby be able to give optimal support.
- The labor ward staff were part of how the birthing room was perceived and by continuous and calm presence, they can contribute to a sense of safety and calm.

## Supplement Material

Supplemental Material, sj-pdf-1-her-10.1177_19375867231163336 - The Birth Companions’ Experience of the Birthing Room and How It Influences the Supportive Role: A Qualitative Study Click here for additional data file. Supplemental Material, sj-pdf-1-her-10.1177_19375867231163336 for The Birth Companions’ Experience of the Birthing Room and How It Influences the Supportive Role: A Qualitative Study by Helena Nilvér and Marie Berg in HERD: Health Environments Research & Design Journal
