# Supplementary material for: The Birth Companions’ Experience of the Birthing Room and How It Influences the Supportive Role: A Qualitative Study
Source: HERD. 2023 Apr 27;16(3):156–67. doi: 10.1177/19375867231163336 (PMC10328140; doi:10.1177/19375867231163336)
Supplement: Supplemental Material, sj-pdf-1-her-10.1177_19375867231163336 - The Birth Companions’ Experience of the Birthing Room and How It Influences the Supportive Role: A Qualitative Study [file sj-pdf-1-her-10.1177_19375867231163336.pdf]

Supplement Material 1

Interview guide used in *The Birth Companion's Experience of the Birthing Room and How It Influences the Supportive Role: A Qualitative Study*

- Can you describe how you perceived the birthing room?
- How did the room affect you?
- Did you feel comfortable in the room?
- Did you feel safe?
- Was there space for you?
- Did the room meet your expectations?
- Did you feel free to adapt the room to your and the birthing woman's needs?
- What types of adaptations did you make?
- Can you describe your role during the labor?
- What was your plan before the labor?
- Could you give the support you had planned?
- Was there anything in the room that helped you give support?
- What did you like the most in the room?
- What in the birth environment was important for you?
- Were there elements of the room you perceived as irritating or distracting?
- Was anything you would have liked missing from the room?
- Is there anything else you would like to add?

Follow-up questions to deepen the responses: What did that mean to you? Can you give an example? Can you tell me more? How did that make you feel?
